# Bullying Trends Inside Sport: When Organized Sport Does Not Attract but Intimidates

**DOI:** 10.3389/fpsyg.2020.02037

**Published:** 2020-12-09

**Authors:** Jolita Vveinhardt, Vilija B. Fominiene

**Affiliations:** ^1^Institute of Sport Science and Innovations, Lithuanian Sports University, Kaunas, Lithuania; ^2^Department of Sport and Tourism Management, Lithuanian Sports University, Kaunas, Lithuania

**Keywords:** bullying, sport, athletes, sports organization, internal environment, coach, bullying behavior, interrelationship

## Abstract

Bullying is acknowledged by scientists as a considerable and still unresolved problem in sport. By triggering stress-related emotions, they determine the behavior of those experiencing bullying and cause various negative effects on their physical and mental health. However, in the presence of the tenacious trend in sports “to put one’s own house in order,” athletes, coaches, teams, and sports organizations themselves often do not emphasize bullying or state that they do not encounter the problem at all, and adheres to the belief that athletes may use negative emotions instrumentally in order to perform tasks given to them more effectively. The aim of this research was to reveal the determinants of the internal environment of sports organizations, causing trends of bullying in organized sport. To achieve the research aim, a qualitative research paradigm was chosen. The empirical study involved eight coaches working in organized sport in Lithuania. The survey was conducted using the semi-structured interview method. Data were analyzed employing inductive content analysis. The presented research results encompass the transcriptions of interviews, which are conceptually divided into three main categories revealing coaches’ opinion on trends of bullying in organized sport, related to the sports organization’s internal environment. Categories identified during the study can be equated to interrelated levels of model of Organizational behavior. The micro level-interrelationships; the mezzo level-sports professionals’ (coaches’) behavior; and the macro level-management of interrelationships. These results revealed which determinants of the sports organization’s internal environment can be favorable for emergence of bullying and its dynamics in both interrelationships among athletes and interrelationships between athletes and coaches. And these trends of bullying, revealed on the basis of the responses of coaches involved in the study, allow us to see harmful principles of coaching, bullying-promoting traditions of team/group leadership, existing in sport, and to predict how this may effect both the athlete himself, his environment and attractiveness of the sporting activity itself.

## Introduction

In the society, sport is often associated with psychological and physical endurance, dramatic and constant struggle, victories. All of this also affects emotional experience of all sport participants: coaches, sports managers, spectators and especially athletes. However, not only positive emotions such as hope, happiness, or joy are experienced in this activity (Jones, 2003; Woodman et al., 2009). Increasing research also reveals the dark side of sport, which usually stays within a separate sports organization, changing rooms of teams or clubs, workouts and which quite often damages the person’s psyche, causing severe emotional consequences. Bullying is one of the subsets of social aggression, which was started to be analyzed more extensively 40 years ago and over the past decade, attracted the increasing interest of researchers in various countries (Smith, 2016). Although research into this phenomenon is conducted in various contexts with research participants belonging to different social and demographic groups, the focus is usually on children’s and young people’s relationships in the school environment (Gentry and Pickel, 2014; Patton et al., 2017). However, one third of bullying occurs outside the school in other social settings too, including the sports environment (Shannon, 2013). Therefore, it is no accident that in recent years, this phenomenon is particularly attracting the attention of scientists dealing with athletes’ interrelationships (Evans et al., 2016; Kerr et al., 2016; Vveinhardt et al., 2016; Fisher and Dzikus, 2017; Vveinhardt and Andriukaitiene, 2017; Nery et al., 2017; Stefaniuk and Bridel, 2018).

However, a clear definition for the term bullying is still missing in the scientific literature. This is related to the traditions of using the concept itself in various languages and cultures, while the very definition is determined by the viewpoint, the circumstances of the emergence of bullying and the contexts of conducted research (Cascardi et al., 2014; Sinkkonen et al., 2014). Nevertheless, it is agreed that bullying is a universal dysfunctional social process (Twemlow and Sacco, 2013) and most definitions of bullying categorize it “as a subset of aggressive behavior that involves an intention to hurt another person” (Phye and Sanders, 2004, p. 4). Such behavior manifests itself as interpersonal aggression or violence, taking many different forms, direct physical violence against the person by shoving, hitting or using psychological violence in the form of name calling, exclusion, humiliation, and rumor-spreading, etc. Such behavioral manifestations most often also damage mental state of the individuals against whom such behavior was directed and due to this cause emotional reactions such as anger, disappointment, fear, anxiety, sadness, shame or demotivation (Jones, 2003; Sampaio et al., 2015). The latter “. may mediate and energize subsequent behaviors” (Deci, 1980, p. 85), including worsening sports activities and drop out of sport (Smith, 1986), and in the long run, may gave negative impact on health and psychological well-being (Ruiz and Hanin, 2011).

Thus, bullying in sport is increasingly recognized as a particularly undesirable expression of aggressive behavior, which causes stress-related emotions, determines behavior of persons experiencing bullying, and causes various adverse effects on physical and mental health. More often, though, regardless of the type of sport or institutional dependence, there is an increasing tendency in scientific literature to analyze aggressive or violent behavior of persons involved in sport, especially in athlete-coach relationships (Stefaniuk and Bridel, 2018), rather than its manifestation through bullying. In any case, bullying, regardless of the stated lack of research (Evans et al., 2016; Fisher and Dzikus, 2017), exists in sport. On the other hand, most of the results and conclusions of conducted research are based on athletes’ opinion (Tamminen et al., 2013; Risner, 2014; Cervin et al., 2017; McPherson et al., 2017), and quite scarcely the phenomenon of bullying is analyzed on the grounds of the position of coaches as the key persons in the formation of interpersonal relationships in sport (Piper et al., 2013; Fathynah and Syahirah, 2015). However, some studies analysing the field of social relationships in organizations emphasize the role of interactions between different levels-micro, mezzo, and macro (Jeurissen, 2005; Appelbaum et al., 2009; Kadic-Maglajlic et al., 2019), although it is not entirely clear how this manifests itself in the sports environment.

Therefore, more extensive research, involving coaches in it, can provide valuable knowledge which could serve as a basis for reviewing the policy of training and leadership of sports teams and bullying prevention which, unfortunately, is often insufficiently effective (Shannon, 2013).

The need for such research is also increased by understanding that athletes’ emotional experiences are related to their performances (Ruiz and Hanin, 2011), which, unfortunately, is often based on the approach of coaches and other organizers of sports activities that athletes may use negative emotions instrumentally in order to perform tasks given to them more effectively. The***aim of this research*** is to reveal the determinants of the internal environment of sports organizations, causing trends of bullying in organized sport.

## Theoretical Background

Bullying as a subset of interpersonal aggression is considered to be a major public problem associated with a series of negative outcomes that eventually affect physical and mental health of victims. It is stated that athletes who have experienced bullying suffer from a lack of motivation, bad mood, fear, headache, they are wearied of chronic fatigue, increased heart rate (Lazarević et al., 2015). Other authors point out victims’ experienced emotional and/or physical suffering (Dussich and Maekoya, 2007), psychosomatic health disorders (Smith, 2015) and other negative consequences for the athlete’s development, health condition and his/her sports career (Lazarević et al., 2015). The problem is also sharpened by quite high probability for the victims to experience bullying further in life, revealed by research (Curwen et al., 2011). Various negative consequences are also experienced by the bullies themselves (Evans et al., 2016), while their behavior can lead to other forms of violence (Fisher and Dzikus, 2017). All the mentioned problems and their emergence in sport are also deepened by tolerating and even promoting aggressive behavior in interpersonal relationships. According to Krishnaveni and Shahin (2014), aggression is part and parcel of any contemporary sport. Often aggression or anger in sport is understood not as behavior that is intended to harm another person who is motivated to avoid that harm (Bushman and Huesmann, 2010), but as an outcome of the competitive struggle, suppressing the opponent’s desire to win or attain the best possible sports results (Oliva-Mendoza et al., 2012; Gencheva, 2015). There is also a trend in sport that alongside with the athlete’s excellence growth coaches increasingly justify athletes’ aggressive behavior that often manifests itself as anger (Loughead and Leith, 2001; Maxwell, 2004). As a result, aggressive acts repeat and other athletes, watching them, start behaving aggressively as well (Sacks et al., 2003). Therefore, we can speak about certain existing trends of perceiving aggression and anger in sport, which directly and indirectly promote and maintain bullying in athletes’ interrelationships.

Data on links between aggression or anger and bullying in the sports environment are so far controversial. On one hand, it has been noticed that sport participation is the activity where the person’s aggressiveness may reduce (Shachar et al., 2016), while sport may become a protective environment that helps to protect oneself from various subsets of aggression, including bullying (Collot-D’Escury and Dudink, 2010; Kentel and McHugh, 2015). The results of other studies show that the influence of participation in sports activities on bullying behavior is not as positive as we can expect (Melim and Pereira, 2013). This means that the sporting activity itself does not eliminate the risk of bullying behavior in the sports environment. This is confirmed by still scarce studies, but their results are often influenced by chosen research methodologies or employed instruments (Fisher and Dzikus, 2017), while the fully unpurified conception of bullying causes difficulties in recognizing this phenomenon (Mishna, 2004).

Studies show that the prevalence of the bullying phenomenon in sport may vary depending on the target group. For example, Nery et al. (2017), who have studied 1458 male adolescent athletes from nine sports in Portugal, found that 10 percent of the athletes reported as being victims, 11.3 percent as bullies, 34.6 percent as bystanders and 44 percent of respondents did not report bullying. The study conducted in Canada by Evans et al. (2016) showed that bullying had been experienced by a slightly larger percentage of research participants; i.e., 14 percent of adolescent athletes. Research conducted in Lithuanian sport revealed even greater levels of bullying. The research conducted by Vveinhardt et al. (2016), dealing with bullying and harassment cases in the teams of Lithuanian schoolchildren’s basketball league, identified that one quarter of all 14–18-years-old athletes who participated in the study had experienced bullying and harassment. A significantly higher number of bullying victims was identified among elite female basketball players: 32.9 percent (Vveinhardt and Andriukaitiene, 2017). The frequency of 50 percent of homophobic bullying was identified in the United Kingdom, which occurred both on the sport field and in the changing rooms (Brackenridge et al., 2007).

The existence of bullying in sport and its specificity is also stated in the results of qualitative studies. For example, Tamminen et al. (2013), who investigated the situation among elite female athletes, found that bullying victimization from teammates was observed in sport. Other studies disclose not only peer bullying, but also draw attention to the coach as a bully (Cense and Brackenridge, 2001; Peltola and Kivijärvi, 2017), while relational aggression is the most frequently reported form of bullying (Kerr et al., 2016). Hence, bullying can acquire not only horizontal (athlete-athlete) but also vertical (coach-athlete) character. However, the results of single studies are not sufficient to provide a comprehensive picture of the extent of the problem in different sport branches in order to distinguish causes and risk factors determining bullying behavior in sport. This means that there is still a lack of comprehensive data needed to draw up bullying prevention and intervention programs. This problem is also confirmed by Stefaniuk and Bridel (2018), who have analyzed how Canadian national sport organizations addressed peer-to-peer bullying through policy.

Perhaps it is hardly possible to fully avoid physical and psychological harm in sport (Slobounov, 2008), but situations where athletes are unable to recognize bullying (Lazarević et al., 2015), do not realize that they are the victims of abuse and, having encountered coaches’ negative behavior, are inclined to think that they rightly deserve punishment (Lazarević et al., 2014), should not occur. That is, certain negative actions are often perceived as a certain phenomenon that is “normal” and tolerable in sport. Therefore, the problem also arises due to violence used by the coaches (Wilson, 2017) or his/her bullying behavior (Cense and Brackenridge, 2001; Peltola and Kivijärvi, 2017), identified in research. In this context, it makes sense to continue research to evaluate how adequately sports organizations are prepared to respond to bullying in sport (Slobounov, 2008; Vardanyan and Ruskina, 2013; Mountjoy et al., 2016). All the more so that in the context of organizational behavior, according to Ashkanasy and Dorris (2017), every level-individual (micro), group (mezzo) and organizational (macro)-affects striving to create a healthier and more productive environment, to ensure psychological well-being and satisfaction of members of the organization (Grobler and Joubert, 2020), and in the opinion of Privitera et al. (2015), to become a prevention of violence. This is also stated in the classic ecological theory that is often used as a basis for analysing risk and protective factors related to involvement in bullying at a young age (Espelage, 2014). In this case, the interaction between the components of the close environment-the micro system is called the mezzo system and can provide insights into how the interactions between different systems can affect the experiences of bullying (Espelage, 2014; Cross et al., 2015; Thornberg, 2015).

## Materials and Methods

In order to answer research questions, the empirical study was constructed, employing the qualitative research strategy, which provides a possibility to view of the problem from the holistic standpoint, focusing on unique human experience in the aspect of the analyzed phenomenon, and to understand its peculiarities. The study is grounded on a constructivist-interpretive paradigm (Creswell, 2007). According to it, there is less focus on phenomena in themselves and more interest in how the phenomenon under analysis is seen (Harper, 2011); i.e., the approach is followed that reality is perceived as a human construct formed from the research participant’s cultural and personal life and does not exist without it. Meanwhile, since the research participant is part of the same reality, his/her and the researcher’s method of interpreting this reality are constructed (Charmaz, 2014).

The study was conducted using the qualitative content analysis approach which is extremely well-suited to analysing data on the multifaceted, sensitive phenomena (Elo and Kyngäs, 2008). This is a method that consists of three main phases: preparation, organization, and reporting of results, where the first, preparation phase consists of data collection method, sampling strategy, and the selection of a suitable unit of analysis; the organization phase includes open coding, creating categories, and abstraction and reporting phase, in which results are described by the content of the categories describing the phenomenon using a selected approach (Elo et al., 2014).

### Participants

The research sample was drawn up using the criterion sampling. In this case, the research sample selects all cases that meet some criterion and helps to ensure the quality of the research data (Patton, 2002). Research participants were sports coaches who worked in amateur sports organizations and trained athletes representing different age groups-adolescents and young adults (10–29 years old). The sampling criteria for coaches were as follows: representation of all three groups of sports: team, combat and individual sports; popularity of sports in Lithuania according to the data of the Lithuanian Official Statistics Portal (2017); representation of different generations; gender differences. The study involved eight coaches: five men and three women and each of them receive a Code (e.g., 1I, 2I etc.) in the transcribed texts. The age of the target group was from 23 to 65 and the seniority of coaches in full-time jobs was from 4 to 30 years (Table 1).

**TABLE 1:** Characteristics of research participants-coaches.

Coach’s code characteristics	1I	2I	3I	4I	5I	6I	7I	8I
Sports group	Team sports/Basketball	Team sports/Basketball	Combat sports/Wrestling	Team sports/Football	Team sports/Basketball	Combat sports/Boxing	Individual sports/Swimming	Team sports/Handball
**Work experience as a full-time coach**								
Years	18	5	10	4	18	6	30	20

Two coaches from all coaches who took part in the survey train three (5I) and four (6I) teams, other coaches simultaneously train one-two teams or groups. With regard to trained athletes, coaches worked only with girls or young women (1I), only with boys and young men (2I; 4I; 5I; 8I) or trained mixed groups (3I; 6I).

### Data Collection

The research data was collected using the semi-structured interview method. Such type of interview was chosen because of its freedom, immediacy and flexibility; i.e., because of created conditions to change the order of given questions, their wordings, to give additional questions, purposefully orientating the participants in the direction of the research phenomenon and consistently deepening the researcher’s perception of the research object. Besides, this type of interview is based on general guidelines to ensure that all interviewees are subject to similar stimuli, thereby allowing a common base for data analysis (Flick, 2009).

To conduct the interviews, interview guides were prepared following guidelines proposed by H.J. Rubin and Rubin (2011) and had introductory, main and summary questions. The first, introductory section consisted of questions about the backgrounds of participants and were intended for making a contact with the subject. The main part of the interview consists of open problem questions arising from the main themes: the importance of interpersonal relationships in the turnover of athletes (“What trends of athletes’ turnover are observed in sports?”), standards of ethical conduct (“What standards of ethical behavior exist in sport?”), the coach’s observation/awareness and the position of the organization’s leader (management) in cases of bullying-harassment (“What are the coach’s and the organization leader’s (management’s) position with regard to bullying?” Summary questions were directed to address minor uncertainties, additional statements, and advice.

These themes and questions were constructed based on scientific literature related to bullying in a general sense and to the analysis of these phenomena in sport as a guide and on authors’ conducted quantitative study, which has revealed experiences of organized sport participants (*N* = 382) (Vveinhardt and Fominiene, 2019) with regard to bullying and harassment. Prior to final data collection, a preliminary interview guides were pilot-tested on one participant-the coach of team sport. This allowed the researchers to ensure the comprehensibility of the questions given in the main study, stability of data collection and solving potential problems with the interview guide (Sparkes and Smith, 2013). Semi-structured interview guide were prepared and conducted in national language.

### Procedure

The study was ethically approved by Lithuanian Sports University Ethics Committee of Social Sciences. Data were collected in January 2019, employing individual semi-structured face-to-face interviews with coaches. The average length of one interview was from 39:14 min to 1:27:90 min.

Before conducting every interview, research participants’ received in writing an informed consent form, kurioje buvo paaiškinta kaip tyrimo metu jø privacy, confidentiality and anonymity were ensured. The participants were introduced to the principles of usefulness and fairness of the research; the research aim, protection of the collected data and the use of future results were also presented to research participants. Research participants had to give their verbal agreement that they did not mind recording of the interview on a dictaphone. The interviewer would give pre-formulated questions, adding new questions emerging while listening to the participant. Audio-recordings were transcribed verbatim by one of the authors. All features enabling to identify the surveyed coach were removed by giving a separate code to every coach and this code is referred to in the “Results” section Afterward, transcribed texts were sent to coaches to check to ensure that no statements had been misinterpreted or wrongly rewritten (Patton, 2002).

### Data Analysis

Data analysis in this research was executed by using hand/human-coding method, according to a predefined coding scheme (Neuendorf, 2019). The whole transcribed text consisted of 41346 words; i.e., 236226 characters. However, this article presents only that part of research results which pertains exclusively only to the position of the organization and the coach on bullying and harassment issues. The transcribed interview text of this part of the study contains 18,029 words; i.e., 103,983 characters.

Inductive content analysis situated within the epistemological position of social constructivism served as the basis for the data analysis (Creswell, 2007). Such method of data analysis was chosen due to of its usefulness for identifying core consistencies and meanings from a large quantity of qualitative data (Patton, 2002). Data analysis was performed in January–February, 2019.

**FIGURE 1:**
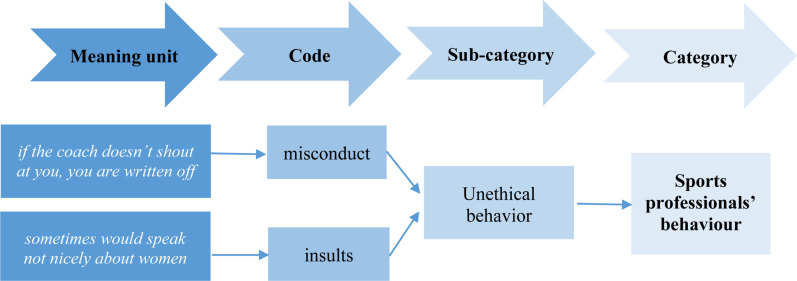
Example of the analysis process from meaning units to categories.

The qualitative content analysis was performed according to the following order: (1) choice of meaning units for the analysis, (2) immersion into research data, (3) open coding, (4) categorization, (5) abstraction, (6) preparation of the research report (Elo and Kyngäs, 2008). In the first stage of data analysis, authors independently read all original transcripts and divided the text into smaller meaning units: the constellation of words or statements that relate to the same central meaning (Graneheim and Lundman, 2004). Afterward, the “open coding process” was carried out; i.e., each identified meaning unit was marked with a code that can be understood according to the context. Based on the study design, codes were generated inductively. In the next stage, subcategories and categories-groups of content that shares a commonality-were distinguished (Figure 1).

Here, at varying levels of abstraction, subcategories were both sorted and abstracted into the category or some categories were broken down into subcategories (Graneheim and Lundman, 2004). A total of fifty-seven codes emerged from the initial coding of data. Analysis generated twelve subcategories and three categories.

The study was conducted following the principles of anonymity, confidentiality, participant security, voluntariness, and authenticity of the research data (De Vos et al., 2011; Leedy and Ormrod, 2014). That is, research participants were assured that the data collected during the data-collection would not reveal their identity: name, surname, workplace or other identifiable information (De Vos et al., 2011). Research participants were also thoroughly acquainted with the purpose of the research, the course and content of the research, and were informed about the use of research data in publicizing the obtained results. Research participants took part in the study voluntarily and were given the opportunity to withdraw from the study at any time (Leedy and Ormrod, 2014).

In order to ensure the trustworthiness and credibility of the study and to reduce the risk of bias, all researchers were involved in the analytic process, the topics purified were comprehensively discussed, and the interpretations provided were based on exhaustive quotations. Besides, detailed interview guides prepared prior to the study ensured that the same questions would be given to investigated persons, this way avoiding interview bias.

The research data collected were not modified, corrected, and were accepted as valuable data that could affect any study outcome unforeseen prior to the study.

### Methodological Rigor

Every qualitative study and its findings should be of high quality. To achieve this, qualitative research must be rigorous (Tracy, 2010), that is to say, rigorous methodological procedures should be used. One of the commonly used criteria for the qualitative research is trustworthiness, as it allows to determine if research findings are actually trustworthy. In our study, trustworthiness and its different aspects such as credibility, dependability and transferability where considered to ensure that the research findings were of high quality.

Credibility is related to the study itself and refers to confidence in how the data obtained during the research and the data analysis process correspond to the selected research. Primarily, the credibility of this study can be proved by the selection of the research context, participants and the approach to gathering data (Graneheim and Lundman, 2004).

Research participants chosen in this study were interviewees with different perspectives, which contributed to richer variations of the phenomena under study. The semi-structured interview as the most appropriate method of data collection enabled to collect the necessary amount of data that is required to answer the research question in a credible way (Graneheim and Lundman, 2004). The fact that the research data were analyzed by two researchers separately and that full agreement on data grouping was reached only after critical discussions, while comprehensive quotations were provided in the report for the purpose of supporting the interpretations, enable to state that the findings represent a credible interpretation of the data (Thomas and Magilvy, 2011). Analysing the research data, it was sought to ensure that the chosen most suitable meaning unit would be neither too broad nor too narrow and the example of the analysis process from meaning units to categories, given in the study (see Figure 1), could facilitate judging credibility of the findings. The literature review performed in the study allowed to evaluate compatibility between the research findings.

Another aspect of trustworthiness is dependability. That aspect is an assessment of the quality of the integrated processes of data collection, data analysis, and theory generation and how well the research findings are supported by the data (Thomas and Magilvy, 2011). In the study several strategies were employed to address these issues.

The use of the purposive sampling strategy ensured appropriate choice of research participants who have diverse experience, which led to rich, thick and detailed description of the phenomena. Researchers’ deep understanding of the field of study before conducting the interviews and remaining “outsiders” facilitated acceptance and rapport with coaches during the interviews. Prepared and tested research guidelines enabled to ensure that participants were asked the same question to attempt to prevent bias between interviews (Sparkes and Smith, 2013). Seeking to ensure consistency during data collection, the period of months was chosen, considering saturation of the data collected in order to avoid extensivity of data. All transcribed texts are stored by researchers as part of the audit trail.

One more aspect of trustworthiness is transferability, which is related to the approach that the findings of the conducted research can be applied in other settings or groups. However, only readers of the study can make a decision about transferability of findings to another context (Graneheim and Lundman, 2004). To this end, the literature review and the discussion part contain detailed information on the research context.

## Results

Content analyses of interview transcripts were conceptually grouped into 12 subcategories and 3 main categories revealing coaches’ opinion regarding elements of the internal environment that exists or is being formed in organized sport, which can be favorable for emergence and dynamics of bullying (Table 2).

**TABLE 2:** Trends of manifestation of bullying in organized sport.

Category	Interrelationships	Sports professionals’ behavior	Management of interrelationships
Sub-category	“Conveyor” principle	Unethical behavior	Refusal/transfer of responsibility
	Pursuit of the “collective good”	Systems of punishments	Ineffective staff improvement activity
	Athlete’s personal qualities	Denial of bullying	Uncertainty of rules
	Bullying as “natural selection”/expulsion from the team	Changing the connotation	Limited understanding of bullying management

The analysis of responses shows that the category “Interrelationships” encompasses the attitude to the athlete from the perspective of interpersonal relationships, existing in sport, which encourages greater turnover of athletes. This category is detailed by sub-categories such as “Conveyor” principle, Pursuit of the “collective good,” Athlete’s personal qualities, Bullying as “natural selection.” The names of some of the sub-categories are expressed in metaphors, this way emphasizing the long-standing traditions of the attitude. The category “Sports professionals’ behavior” is associated with behavioral patterns of sports organization’s employees, which are favorable to the existence of bullying. This category consists of sub-categories such as Unethical behavior, Systems of punishments, Denial of bullying and Changing the connotation. The third category “Management of interrelationships” reveals managerial practices within sports organizations, contributing to escalation of bullying or making prevention ineffective. This is related to Refusal/transfer of responsibility, Ineffective staff improvement activity, Uncertainty of rules and Limited understanding of bullying management.

### Interrelationships

The coaches’ demonstrated attitude came to prominence, showing that they could treat the athlete as a “product” that they use in their work activities to reach the organization’s or their aims:

*“…first-graders come, learn for four or six years and they leave. Again, I get novices and my vicious circle. There is natural selection in my work. I raise the product, give it away, take the new product again, again I grow it, give it away. Everything goes naturally*” (1I).

In other words, the depersonalized attitude toward the athlete comes to prominence, and the coaching process turns into the implemented “conveyor principle.” Athletes change each other, and such attitude eventually becomes more and more acceptable personally to the coach, because personal responsibility for the process of athletes’ drop out disappears. The explanation of participant 5I shows that during training, coaches are not that much encouraged to look for specific reasons but to justify the turnover as the “natural process”:

“…*because you would start looking for something that you might be doing wrong that some team members leave, others come”; “* < *…* > *but both in all workshops and trainings, it’s really natural so-called change: “don’t worry, coaches”; “* < *…* > *this* [withdrawal] *is based on curiosity, interest in sport types at a certain age period: boys still want to try out one or another area until they find their favourite area and the like*” (5I).

Such approach, like a refrain, is also repeated in the responses of participants 3I and 4I, suggesting a well-established simplified thinking tradition, which may hinder an adequate evaluation of the influence of bullying on athletes’ decisions to leave the team. The athlete’s depersonalization is related to the logic of the “collective good” -yet another trend of the “simplified” approach to coaching and relationships in the team. This trend is most clearly revealed in the explanation of participant 2I:

“…*there were several withdrawals during this year, when you see that if you withdraw that player from the team, the collective will become more harmonious and, to sum up, the results will be better. You have to do this because the player does not tune to the team, he is separated from all, doesn’t integrate into the collective and you see that it will sooner or later influence the results on the court…”* (2I).

The participant transfers the responsibility for “integration” to the athlete himself. Creating “good” for the collective, this is most often associated with the image of the strong team or winner, and the latter is inseparable from understating of the weaker. The coach, who can make decisions as to who will stay for training with him, plays a considerable role in this change. This may be influenced by the subjective assessment of the young athlete, performed considering his talents: *“*… *either he must be that super-boxer, so that I am guaranteed that he will become the European champion, then okay. Then you would try to manage him*…*”* (6I). That is, the presumed “value” of the athlete determines the relationships with the athlete; therefore, at the same time, there appears the danger of indulging the promising athlete for his/her behavior. Moreover, the coach’s attitude may also be influenced by the opinion formed among athletes about individual team members, as illustrated by statements of participant I1:

“…*because they are weaker. Come, see that oh, I took the wrong road, I still have to learn a lot here. And gets that kind of response, that, well, coach, it’s not that we’re disturbed here, but. And she sees, I got into the wrong medium. I need a step lower or, well, I need to withdraw from here. So, that’s it. Natural here, I think, selection. Natural. Weaker is weaker…*” (1I).

In this process of change, in the coach’s opinion, an important role is played by the athlete’s personality traits determining resistance to emerging or existing destructive relationships in sport. Only the strongest athletes firmly remain in the athlete’s role; therefore, coaches particularly emphasize athletes’ psychological weakness, which often leads to departure from the team *“. he isn’t physically weak, and he is a gifted child, but he is psychologically weak. He is very weak psychologically”* (6I). There is an attitude that having encountered bullying, only psychologically firm athletes can remain, in whose behavior coaches envisage manifestations of aggression that are desirable to the athlete:

“…*when the child comes to the first training session, most often, if he is more sensitive, he is sneered at, of course, sneering is not that sharp, maybe he may not come to the next training session, but the child who is slightly stronger, as I say, psychologically, he comes and, as I say, this is such slight bullying. naturally selects those children in martial arts, it is not a bad thing this because the child must have some character traits*-*of the fighter, if he surrenders, then…*” (3I).

In other words, bullying tends to be justified and treated positively as “natural selection”. In other words, bullying tends to be justified and treated positively as “natural selection”. The turnover may be also caused by degrading mastery in the presence of other persons: “. *finally, shouting that you can’t, don’t do, some sort of shouting at some other leader, teammate can expel him instantaneously*” (5I). That is, this negative communication is used instrumentally, in order to expel the athlete from the team.

### Sports Professionals’ Behavior

Coaches’ experiences related to bullying in sport also highlighted the specific behavior of coaches or sports organization’s leaders, determined by sports context. However, although the behavior “favorable” for manifestation of bullying in sport can be described as intolerable, it exists in sport, and top managers of sports organizations get involved in it. This is revealed by the sub-category “Unethical behavior”. I1 participant’s answer gives prominence to the opinion that the shouting coach is the norm: *“. and how we say: if the coach doesn’t shout at you, you are written off”*, which implies that such coach’s behavior is permanently demonstrated during training sessions and competitions. The top managers of the organization himself demonstrates unethical behavior, manifesting itself by disrespect to people around him:

“…*and sometimes I didn’t really like him (top manager) that he would use some kind of swear words in the presence of those, my teenagers. So, he would use these curse words and this way sometimes would speak not nicely about women*…” (7I).

On the one hand, this way, such offensive utterances form an intimidating environment and are legitimized as a certain norm in the eyes of athletes; on the other hand, the message is sent that similar attacks from team members will not be addressed at the management level. Intolerable behavior in sport is also revealed through the “system of punishments” existing in every team. The system of punishments and rules can be dictated by the coach or his delegated team captain, and they may be related to the efficiency or usefulness of the team member’s participation in the workouts and the play. The statements of participant 2I shows that both rulemaking and decisions are left “to themselves”, trusting formal leaders:

“…*these rules are formed by team coaches, the team captain and there already they, after that, in the collective those punishments, if they are late or something, they settle themselves*” (2I).

However, the punishments applied are not defined in the written form but performed at the discretion of the coach:

“…*because of bad behaviour just can be expelled from the team, suspended within the limits of a certain norm, certain sanctions in the team’s internal management: to miss the match, miss the tournament and the like. Bad behaviour, disrespect to the coach*-*please leave the gym, from the team go home, out of the gym. Comes, if apologizes, discussion, etc…*” (5I).

It is stated that punishments are applied for breaches of public conduct rules, but it is significant that sanctions are differentiated by age: age gives the privilege not to follow rules: *“. well, there you seat him for five to ten minutes to watch the workout. Such punishments, gentle, non-physical.* < . > *Of course, older is older, but in the team of small ones very strictly with that”* (4I). In addition, the choice and application of punishments depend on the situation and are ambiguous. For example, the athlete who violated rules misses only that match that seems unimportant to the coach. As shown by the explanation of 5I, there are no rules and the system of punishments clearly described for everyone:

“…*to exclude players from the team composition in the unimportant match, not to allow to play, to see what the player likes a lot or is waiting for some tournament or other things. preventive these things. It works very well. Especially leader players who, you see that they love that sport but violate ethics*” (5I).

That is, more freedom of behavior is given even in negative aspects, especially if the athlete is considered beneficial to the team. This paves the way to the abuse of the existing position. In addition, it becomes clear that the rules of “ethical” behavior apply to athletes only during the training: *“. the children themselves then understand that we will be able to watch, talk to each other afterwards, later”* (4I). This way, unethical behavior is merely transferred from the public to the private space. Another related aspect of the problem is avoidance to recognize the existence of bullying in the team by diverting attention to other teams. This comes to prominence in the response of participant 1I:

“…*but in my work, in my team, there is no such. Such event, cases. No. Maybe you should look in the boys’ group. For sure they curse at each other and everything there*” (1I).

Besides, it is aimed to understate bullying itself, trying to change the connotation of the bullying action *“. of course, sneering is not so sharp”* (3I) or to reduce its significance, relating to age:

“…*in the older age, bullying somehow, maybe sneering exists but it takes a very different form than in children’s sport, where those children react much more sensitively.* < . > *Bullying in the older age greatly alters the form and goes to the background”* (3I).

In other words, bullying among children and young people is treated as natural and it is believed that this is resolved naturally when athletes grow up. This demonstrates that the nature and role of bullying in the sport team are insufficiently perceived and such attitude on one hand, can be the means of disguising incompetence and on the other hand, creates favorable conditions for existence of bullying.

### Management of Interrelationships

The subcategory distinguished in this category is “Transfer of responsibility” indicating the trend to transfer responsibility for the sports training as a psychosocial process of interaction to other persons. Often the quality of emerging interpersonal relationships and its consequences are not important for sport organization management. This is evidenced by the coach’s observations that responsibility for that is given over to others, and, first and foremost, to the coach, and the latter transfer responsibility to parents:

“…*I don’t know in this organization, we, the educators are familiar with these manifestations and preventions, and, I think that management probably trusts us”* (1I). “. *in that period up to the age of sixteen, parents have to bear that burden, because parents primarily must teach that they can’t jeer, can’t jeer at the smaller ones and that they have to show a good example during the match themselves. They can’t shout using swear words at opponents’ children or somehow otherwise. They can’t let children write on Facebook what they want and how they want. It’s purely parental responsibility, because the coach’s job is to train him, parents educate”* (2I).

In other words, the significance of athletes’ negative behavior is perceived, but responsibility is not shared-it is refused by transferring it to others. Therefore, measures that could help to solve the problem of bullying remain unfulfilled. In order to avoid bullying in sport, it is very important to have rules governing sports participants’ behavior in the organization and follow them. The sub-category Uncertainty of rules reveals how and what rules are developed in organized sport; i.e., in a particular sport type or organization. Often there are no written rules of conduct, which possibly gives freedom to behave negatively, because “. it’s important that only the goal is achieved” (1I). In such case, the rules are formed by every coach personally “. and they exist everywhere” (6I). Hence, so many coaches, so many rules. The absence of uniform written rules usually allows to interpret them freely, especially when the regulatory power is delegated to athletes depending on the status defined by their age:

“*Well, first of all, it is I who sets those rules. I tell how they should behave during training sessions, matches regarding respect to the opponent, to the judge*” (1I). “. *what is not permitted, of course, only the coach. The coach stops. Well, actually, among those older ones there are cases when they are stopping each other: “Will you stop here? What you, what are you doing here?” There are cases when he comes after school tired, tired of everything, and you see that he is in a bad mood, so if you. if you still him*… *If in addition to that you say something, then the reaction becomes totally angry, then I see that these older ones themselves are already halting: “Will you finish, what’s wrong with you, go home to rest, you’ll come the next day*” (8I).

Absence of objective rules defined by the organization paves the way to subjective use of power, which does not guarantee impartiality and fairness. While coaches are creating rules, a peculiar philosophy also comes to prominence, dominated by “. thinking that there must be tough environment in the team so that they (athletes) harden and so that afterward they show the result on the court” (2I). In other words, violent actions can be perceived as a pedagogical measure. Such environment naturally allows the formation of negative relationships grounded on bullying of the weaker ones. This is a stagnated culture of athletes’ development:

“…*no coaches have changed for almost thirty years. And you have already seen many generations of these learners. And now you see those generations*” (2I).

The latter can be associated with coaches mentioned insufficient staff improvement in the sports organization (distinguished sub-category “Ineffective staff improvement activity”):

“…*educators are given more attention, all educators, but in physical education, sports centres such as seminars or still something else, no, there are not that many tools as at school*” (3I).

This leads to the situation in which the ability to envisage bullying taking place among athletes and effectively manage them becomes the matter of every coach. However, the prerequisite for that is the very coach’s wish: *“. we are just improving ourselves if we want. We’re raising our qualification. Listening to seminars or lectures on that subject. But such, well, from management, that word special, there is no such really. Nothing like gathering sports centres and conducting something like that, no”* (1I). However, the “wish” highlighted by 2I is, unfortunately, not equally strong with all coaches:

*“…actually, all that burden to avoid bullying depends on the coach’s competencies. And there are various coaches. Coaches are of one or another type, and I really know that they make the environment favourable to bullying, but you can’t solve somehow without proofs. Such mentality, such thinking that the environment in the team must be tough, so that they harden, so that they then show the result on the court*…“ (2I).

In principle, this corresponds to the stereotype in the society that the army or sport, where relationships grounded on bullying exist, is a kind of “school of masculinity.” In organized sport, this also determines coaches’ individual understanding of bullying management. Coaches’ willingly presented personal insights on bullying management methods disclose a distinct lack of knowledge. It is stated that bullying prevention requires educational measures, but it is evident that they are not guided by such measures in their team and do not familiarize their trained athletes with that, as shown in the explanation given by I1:

“…*well, only by education, of course. By explanation about consequences, what may be bad. Well, that, well, not only bad for you, but that you, that you were offended by someone, but for that offender too, he also has to feel bad. Should feel because he is also problematic. I don’t know, I relate this to education and explanation. And that well. well, that’s the coach’s key role. The coach’s, parents*” (1I).

According to coaches, it is necessary to involve parents, although it is recognized that there is no clear system and knowing how to do this: *“. a personal conversation with the athlete, being not afraid to involve parents too, if they are willing to speak with you”* (8I). Naming other measures to guide bullying prevention, it is attempted to imply that these may be talks taking various forms *“. we need to talk, for example, to them both together and with each separately”* (4I) or organized joint events *“.some celebrations should also be arranged”* (4I). During the interview, the coach’s understanding about bullying as intolerable behavior comes to prominence (sub-category “Limited understanding about bullying management”); therefore, based on the authority right given by the coach’s role, attempts are made to simulate preventive actions:

“…*we, how, say, at the micro level, coaches-team leaders are responsible for that prevention, for those individual programs. Each of us can prepare them, action plans individually: we will organize events, social and the like to reduce bullying, some meetings with athletes and the like…*” (5I).

Coaches do not underline the organization’s responsibility for interpersonal relationships in the sport performance, besides, efforts to hide emerging conflict situations from the organization’s management come to prominence.

“…*I really can’t answer what is going on at the sports school’s level. I haven’t encountered that, I try to figure it out myself, within the team, so somehow I don’t even know if there is anything there…*” (8I).

This creates a vicious circle where the organization does not care about the development of coaches’ competencies in the field of bullying management, does not establish uniform rules, transferring responsibility to the coach, who is interested in hiding incidents from the organization’s management.

## Discussion

**FIGURE 2:**
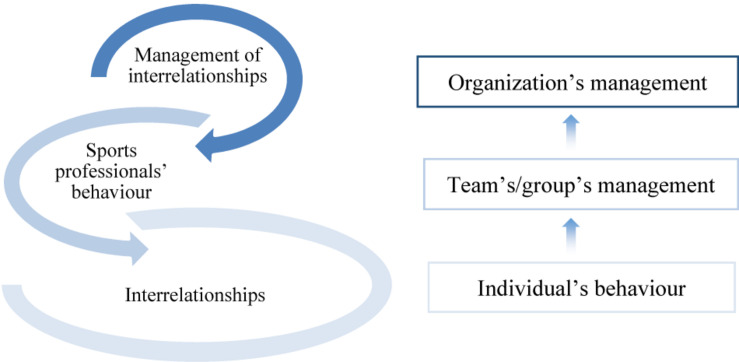
Determinants promoting/supporting bullying at different levels of the intrinsic environment of the organized sport.

This study confirms that the quality of coach-athlete interpersonal interactions depends on a broader organizational context (Lazarević et al., 2015; Stefaniuk and Bridel, 2018; Parent and Fortier, 2018; Kerr et al., 2019). The results of the study enabled to distinguish the determinants promoting bullying in sport, which can be relatively divided into groups covering three different levels (Figure 2).

These three interrelated levels that crystallized during the analysis respond to a widely accepted model of organizational behavior, which can help to discover solutions enabling to improve the performance of the organization as a whole (Ashkanasy and Dorris, 2017). In addition, the above-mentioned three levels are also analyzed in various business ethics (Jeurissen, 2005; Kadic-Maglajlic et al., 2019) and organizational leadership (Kollenscher et al., 2018) issues, which is also relevant in the context of this study.

At the first, the micro level, attention is drawn to the individual’s behavior, and specific interrelationships along with the resulting athletes’ turnover in teams are revealed. This level is also supported by the very conception of bullying, which reveals that bullying occurs when individuals establish permanent relationships. However, the social context is particularly important for that as well, as without it, bullying acts are not possible (Cook et al., 2010). Although a considerable share of scientific literature states the benefit of sport as a social context for the young athlete’s versatile development, not all sport participants state having gained positive experience (Bruner et al., 2017). The existing coaches’ attitudes toward athletes, grounded on the pursuit of the highest possible athletic performance, and the desire to educate athletes who are psychologically strong and only this way able to achieve high athletic performance, analyzed in the study, allowed to distinguish several key determinants of athletes’ turnover due to existing interrelationships: coaches’ behavior directed toward maximum performance, which is logically related to the search for the best athlete and competition-based relationships between teammates.

In the first case, the coach’s behavior is oriented to the search for the most useful athlete, pushing out unsatisfying candidates. To achieve this, the coach uses both psychological impact measures to get rid of the unwanted athlete and the dynamics of bullying taking place in the team, which he implicitly approves of. Approval comes to prominence in the attempts to understate the harm of bullying in stereotypical attitudes, justifying the winner’s priority. In this context, the winner’s image is associated with the so-called psychological strength, which is perceived as the competitors’ psychological crushing or the ability to resist such crushing. In the second case, unfair competition among athletes, seeking to push out the unwanted person, shows up.

Therefore, the constant turnover of athletes, noticed at the micro level, can signal bullying-related problems existing in the team. Other studies analysing key determinants of drop out of sport also distinguish behavior of coaches and teammates (Fraser-Thomas et al., 2008; Hassan et al., 2017). Seeking victories and educating psychologically strong athletes, coaches do not avoid shouting, shoving, hitting them (Narwal, 2014; Peltola and Kivijärvi, 2017), while athletes interacting with teammates who are weaker or whose sports mastery is lower do not shy away to mock at them (Steinfeldt et al., 2012). This trend is also particularly supported by the approach that negative behavior in sport is simply mandatory in order to achieve results (Stirling and Kerr, 2014), and often it takes the form of bullying (Fisher and Dzikus, 2017). However, our study accentuates a dangerous approach that conflicts taking place at the micro level, including bullying, are perceived instrumentally as processes that are natural and useful for sport, enabling to get rid of unwanted persons “naturally.” Similar trends are also recorded in other studies emphasizing that the social context plays a fundamental role in the dropout of the sports process (Sarrazin et al., 2002). Unfortunately, only a few studies link the athletes’ dropout process to the existence of bullying in sport and analyze its causes (Baiocco et al., 2018).

The second, mezzo level is directed to the team’s/group’s management that is implemented through sports professionals’ (coaches’) behavioral strategies. They are entrenched by an authoritarian governance style and require a justification for rude behavior. The learning theory explains why bullying can remain viable in the organizational environment (Altman, 2010), and the striving to justify the coach’s unethical behavior not only hinders change in the situation but can also promote a universal denial of bullying.

Such behavior of the sports professional can be explained by the power of authority given to him/her; i.e., coaches are considered “.an authority figure and often must be firm and exercise that authority” (Narwal, 2014, p. 112), but their lack of competence can lead to unsuccessful prizewinning relationships or ineffective caring and helpful relationships (Jowett, 2005). This seems to be not a problem of individual sports organizations-it can be treated as a part of a flawed tradition. Although the links between the coach’s unethical behavior and bullying in the sport context are not often analyzed, research in other contexts such as the academic environment or workplace reveals such links (Pörhölä et al., 2006; Aleassa and Megdadi, 2014) and states that only the creation of the supportive and safe environment through ethical communication can reduce bullying. Still, this study shows that coaches acting at the mezzo level are creating a specific environment of athletes’ interrelationships, which is based on the traditions existing in sport and individual intentions of coaches themselves. Bullying in sports is a culturally (through coaching traditions) entrenched problem, which is erroneously understood in the coaching practice as bottom-up practice: athletes-coaches, when coaches perform only the function of controlling athletes’ behavior, where the perpetrator is the athlete, his character and his closest environment.

Finally, the third, macro level shows the lack of management practices of the sports organization implementing organized sports activities, related to interrelationships management. Although recent research emphasizes the problem of bullying in sport and the need for effective prevention and intervention (Mountjoy et al., 2016), leaders of sports organizations often do not give prominence to it, which determines the absence of appropriate bullying response protocols (Shannon, 2013). Our research results demonstrate that the coach being at the mezzo level of the sports organization and directly encountering athletes’ interpersonal relationships is a key person who can make a significant impact on bullying prevention and apply intervention measures. However, he requires support at the organization’s macro level. However, although coaches formally act in the sports organization, the latter tends to delegate all responsibility regarding interrelationships to coaches. Often, coaches tend to delegate responsibility for athletes’ behavior and discipline in the team to their parents or other team leaders. This trend prevents effective resolution of bullying-related problems, since, according to Wilson (2017), only regular cooperation between sports organization’s staff and athletes, their families or friends can contribute to the solution of the problem.

The lack of interpersonal relationship management practice is also determined by the ineffective coach development system, the gaps of which are also reasoned by the lack or even absence of coaches’ knowledge related to the protection of rights of children playing sports, found by research (Eliasson, 2015). A better understanding of the topic of bullying by coaches of athletes can ensure effective prevention of bullying (O’Neill et al., 2014; McCloughan et al., 2015; Kowalski, 2017). However, the existing position of the sports organization with regard to staff development, continuing to prevent coaches from gaining knowledge and abilities to identify and manage bullying, leads to every sports participant’s individual understanding of what bullying is, how it should be managed and whether it should be managed at all.

## Conclusion

This study deepens the understanding of the reasons for the viability of bullying in the sports organization and explains its durability from the perspective of the well-established coaching culture. Therefore, the evaluation of the conditions that support and promote bullying in the sports environment enables to take further actions to ensure the environment that is safer for athletes and more favorable for their training.

Creation of such environment requires to pay attention to the critical factors manifesting themselves at three levels. The constant change of athletes, noticed at the micro level, can signal problems existing in the team, which should be divided into two generalized groups: unfair competition among athletes, seeking to push out the unwanted person, and the coach’s behavior orientated to the search for the most useful athlete, pushing out unsatisfying candidates. In this case, the coach uses both psychological impact measures to get rid of the unwanted athlete and the dynamics of bullying taking place in the team, which he implicitly approves of. Coaches acting at the mezzo level are creating a specific environment of athletes’ interrelationships, which is based on the traditions existing in sport and individual intentions of coaches themselves. Along with that, the research discloses the lack of ethics and athletes’ interrelationships management competencies, which is not eliminated by the organization keeping itself aloof. Although coaches formally act in the sports organization, their expressed position and the lack of regulation signal the peculiarly existing autonomy at the mezzo level, tolerated by the organization’s aloof management, which assesses the team’s performance as a final result of activities but does not take responsibility for internal processes.

In summary, it can be stated that the results of the study pose managerial and ethical challenges, as organizations must change well-established attitudes and take responsibility for the creation of the safe sports environment that becomes a priority for coordinated actions of the coach and the organization’s management.

The insights of this study can serve as a basis for more detailed research on the bullying phenomenon in sports organizations. This study highlighted the use of bullying by coaches as an illicit instrument seeking to eliminate unwanted athletes; therefore, in the future, more detailed studies should be conducted to explain the causes of this trend, related to the regulations of team formation, sports organization’s management and the principles of submitting appeals regarding coaches’ behavior. It also makes sense to further explore why athletes who have experienced team members’ and coaches’ unethical behavior withdraw and how the organizations’ anti-bullying policy influences that. The study was conducted in Lithuanian sports organizations; therefore, in the future, it would make sense to repeat it in other countries, highlighting cultural variables.

## Data Availability Statement

The datasets presented in this article are not readily available because they are collected in Lithuanian. Requests to access the datasets should be directed to author of the article JV, jolita.vveinhardt@lsu.lt.

## Ethics Statement

The studies involving human participants were reviewed and approved by the Lithuanian Sports University Ethics Committee of Social Sciences. Written informed consent for participation was not required for this study in accordance with the national legislation and the institutional requirements.

## Author Contributions

Both authors participated and contributed in study design, data collection, analysis and interpretation, writing and original draft preparation.

## Conflict of Interest

The authors declare that the research was conducted in the absence of any commercial or financial relationships that could be construed as a potential conflict of interest.
